# First-Principles Modeling of Nitazoxanide Analogues as Prospective PFOR-Targeted Antibacterials

**DOI:** 10.3390/ijms262311578

**Published:** 2025-11-28

**Authors:** Huda Alqahtani, Islam Gomaa, Ahmed Refaat, M. S. A. Mansour, Raiedhah A. Alsaiari, Moustafa A. Rizk

**Affiliations:** 1 Department of Chemistry, College of Science, King Saud University, P.O. Box 2455, Riyadh 11451, Saudi Arabia; haalgahtani@ksu.edu.sa; 2 Nanotechnology Research Centre (NTRC), The British University in Egypt (BUE), Suez Desert Road, Cairo 11837, Egypt; 3 Spectroscopy Department, National Research Centre, 33 El-Bohouth St., Dokki, Giza 12622, Egypt; am.refaat@nrc.sci.eg; 4 Molecular Modeling and Spectroscopy Laboratory, Centre of Excellence for Advanced Science, National Research Centre, 33 El-Bohouth St., Dokki, Giza 12622, Egypt; 5 Chemistry Department, Faculty of Science, Cairo University, Giza 12613, Egypt; adammidomansour@gmail.com; 6 Department of Chemistry, College of Science and Art, Najran University, Najran P.O. Box 1988, Saudi Arabia; raalsayari@nu.edu.sa; 7 Chemistry Department, Faculty of Science, Suez Canal University, Ismailia 41522, Egypt

**Keywords:** pyruvate:ferredoxin oxidoreductase (PFOR), nitazoxanide, tizoxanide, molecular docking, density functional theory (DFT), QSAR, drug design

## Abstract

Pyruvate:ferredoxin oxidoreductase (PFOR) is a key Achilles’ heel in anaerobic pathogens. We integrate electronic-structure calculations (DFT), cheminformatic QSAR metrics, and residue-resolved docking to distill a concise “recognition code” and translate it into practical design rules. Using nitazoxanide (Nita; ΔG_(bind)_ ≈ −10.0 kcal·mol^−1^) as a well-established reference, productive binding requires a conserved triad: a hydrogen-bond donor addressing Thr-997 and Cys-840, a π–π stack with Phe-869, and a recurrent π–σ contact to Thr-997 that orients the scaffold. Deacetylation to tizoxanide unmasks the phenolic donor and raises local electrophilicity, yet it also slightly loosens pocket packing (−9.6 kcal·mol^−1^). Strategic halogenation introduces a σ-hole interaction near Pro-29, tightening pose geometry without disrupting the donor network; the lead analogue yields −10.1 kcal·mol^−1^, and two others match the reference by preserving the triad and hydrophobic belt. The result is a minimal, testable recipe—retain the phenolic donor, enforce Thr-997/Cys-840 and Phe-869, and add a calibrated halogen σ-hole—offering falsifiable predictions to surpass nitazoxanide and guiding synthesis and biophysical validation in targeted PFOR inhibition.

## 1. Introduction

Nitazoxanide (Nita) and its deacetylated metabolite tizoxanide (TIZ) are broad-spectrum thiazolides with established clinical utility against anaerobic protozoa and enteric pathogens [1], yet their precise structure activity relationships and target-engagement determinants remain incompletely resolved [2]. Converging biochemical and chemical-biology evidence has implicated pyruvate:ferredoxin oxidoreductase (PFOR) as a privileged target in susceptible organisms, consistent with thiazolide activity under low-oxygen metabolism and with ligand recognition features in the thiamine-pyrophosphate/[Fe–S]-dependent active site [3]. Prior studies on thiazolide analogues have largely explored modifications to the salicyl/benzamide and thiazole moieties to tune lipophilicity, hydrogen-bond capacity, and electronic character [4,5,6], and several computational reports have correlated global reactivity indices (e.g., HOMO–LUMO gap, electrophilicity) and surface electrostatics with docking-predicted binding to targeted enzymes such as PFOR [7,8,9,10,11]. However, a unified, mechanistically consistent framework that links quantum-level electronic structure, medicinal-chemistry descriptors, and residue-resolved binding modes across a systematically varied thiazolide series is still lacking. Here, we address this gap by integrating density-functional theory (DFT) modeling, quantitative structure-activity relationship (QSAR) analysis, molecular electrostatic potential (MEP) mapping, and molecular docking against PFOR to interrogate Nita, TIZ, and a focused set of rationally designed analogues bearing electron-withdrawing (e.g., –NO_2_, halogens) and electron-donating (e.g., –OH, –CH_3_) substituents. The central aim is to delineate how substituent-driven changes in polarity, polarizability, softness/electrophilicity, and H-bond donor/acceptor profiles govern recognition within the PFOR pocket, especially the balance between a conserved hydrogen-bonding anchor and apolar/π-driven contacts observed near residues implicated in ligand stabilization, and to translate these trends into clear, testable design rules. Beyond their classical use to modulate lipophilicity and metabolic stability, halogen substituents on aromatic rings can engage in σ-hole-driven halogen bonding with electron-rich protein sites [12]. In heavy halogens such as Cl, Br and I, the anisotropic charge distribution creates a localized region of positive electrostatic potential (the σ-hole) along the C–X bond axis, which can form highly directional contacts with backbone carbonyl oxygens or heteroatom lone pairs, often with geometries analogous to hydrogen bonds [13]. Such halogen bonds are now widely recognized as privileged pharmacophore elements that can enhance affinity and selectivity in protein–ligand complexes without disrupting existing interaction networks [14]. In the context of PFOR, the proximity and orientation of the Pro-29 backbone carbonyl suggest a plausible acceptor for a C–X···O σ-hole interaction [15]. This motivated us to explore haloaryl nitazoxanide analogues designed to position a tunable σ-hole near Pro-29, complementing the established hydrogen-bonding and π-stacking contacts within the thiazolide binding region. By coupling DFT-derived reactivity indices and surface potentials with drug-relevant QSAR descriptors (log P, polar surface/accessible areas, HBD/HBA, and related metrics) and validating the emergent hypotheses through molecular docking binding poses and interaction fingerprints, this provides a coherent, cross-scale rationale for potency-relevant features on the thiazolide scaffold. This combined computational strategy not only rationalizes the distinct behaviors of Nita versus TIZ but also nominates optimized analogues with improved physicochemical balance and predicted PFOR affinity, offering concrete starting points for synthesis and experimental profiling.

## 2. Results and Discussion

### 2.1. DFT Molecular Modeling and Electronic Structure Analysis

**Figure 1:**
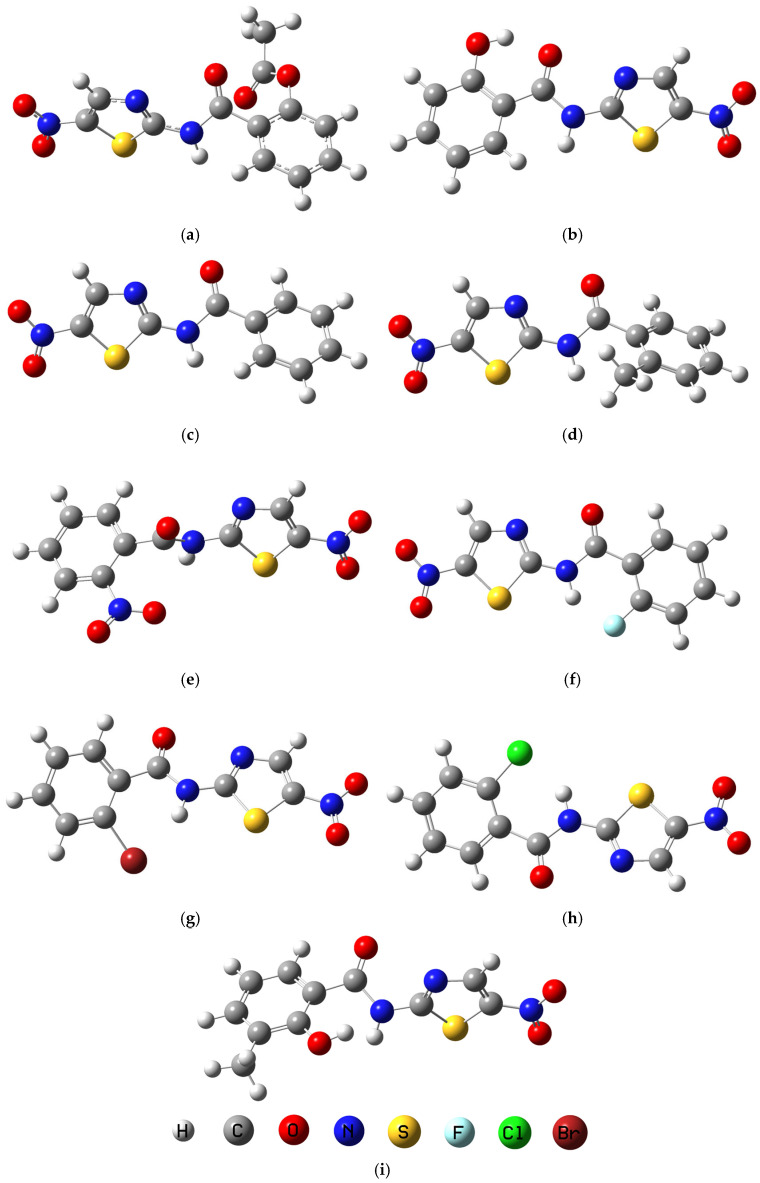
Optimized structures of (**a**) Nita; (**b**) TIZ; (**c**) Mol 1; (**d**) Mol 2; (**e**) Mol 3; (**f**) Mol 4; (**g**) Mol 5; (**h**) Mol 6; (**i**) Mol 7.

Density Functional Theory (DFT) calculations at the B3LYP/6-311+G(d,p) level were employed to gain deep insights into the electronic structures, reactivity, and the intermolecular interaction potentials of Nita, TIZ, and their analogues are demonstrated in Figure 1. The analysis of dipole moments, frontier molecular orbitals, and global reactivity descriptors provides a quantum-mechanical basis for interpreting their observed biological activities.

#### 2.1.1. Dipole Moment and Molecular Polarity

The calculated dipole moments are presented in Table 1. The dipole moment is a critical property influencing a Mol’s solubility in polar solvents and its interaction with biological targets, such as binding to polar residues in an enzyme’s active site [16,17,18]. 

All compounds exhibit significant dipole moments (>6 D), consistent with their polar functional groups (amide, carbonyl, nitro, halogen). The metabolic conversion from the prodrug Nita (7.51 D) to the active drug TIZ (6.39 D) results in a notable decrease in polarity, which may contribute to differences in their distribution and binding. As anticipated, Mol 3, functionalized with the strongly electron-withdrawing nitro group (-NO_2_), possesses the highest dipole moment (9.21 D). Conversely, Mol 7 has the lowest dipole-moment (6.16 D) in the series, suggesting it may possess superior membrane permeability compared to its analogues.

#### 2.1.2. Frontier Molecular Orbitals and Chemical Reactivity

The energies of the Highest Occupied Molecular Orbital (HOMO) and Lowest Unoccupied Molecular Orbital (LUMO), along with their energy gap (ΔE = E_LUMO_ − E_HOMO_), are summarized in Table 2. The HOMO energy represents the electron-donating ability (ionization potential), the LUMO energy represents the electron-accepting ability (electron affinity), and the HOMO-LUMO gap (ΔE) is a measure of kinetic stability and chemical reactivity [19,20].

**Figure 2:**
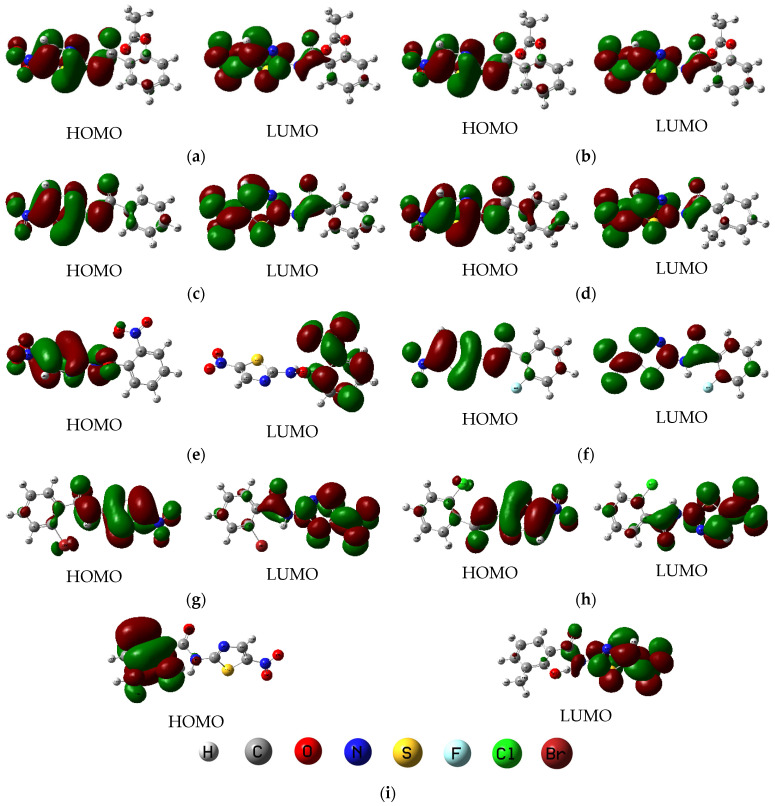
HOMO and LUMO distribution plots of (**a**) Nita; (**b**) TIZ; (**c**) Mol 1; (**d**) Mol 2; (**e**) Mol 3; (**f**) Mol 4; (**g**) Mol 5; (**h**) Mol 6; (**i**) Mol 7.

A smaller ΔE implies higher chemical reactivity and a greater propensity for charge transfer interactions. TIZ exhibits a significantly smaller gap (3.65 eV) compared to its prodrug Nita (4.15 eV), indicating that the active metabolite is inherently more reactive, which may facilitate its interaction with biological targets. This trend is even more pronounced in Mol 7, which has the narrowest gap in the series (3.57 eV), suggesting it could be the most reactive analogue. The HOMO-LUMO plots in Figure 2 reveal that the electron density in the frontier orbitals is predominantly delocalized over the central thiazole and aromatic rings for all compounds, indicating that this core scaffold is the primary locus for electronic transitions and charge-based interactions.

#### 2.1.3. Global Reactivity Descriptors

Using the HOMO and LUMO energies, key global reactivity descriptors were calculated (Table 3) within the framework of conceptual DFT [21]. These include ionization potential (*IP* = −*E_HOMO_*), electron affinity (*EA* = −*E_LUMO_*), chemical potential (*μ* = −($\frac{IP\;+\;EA}{2}$)), hardness (*η* = $\frac{IP\;-\;EA}{2}$), softness (*S* = $\frac{1}{\eta }$), electrophilicity index (*ω* = $\frac{\mu^{2}}{2\eta }$), and electronegativity (*χ* = −*μ*) [22].

Inspecting the value of chemical hardness/softness, TIZ and Mol 7 show lower hardness (η = 1.83 and 1.79 eV, respectively) and higher softness (S = 0.55 and 0.56 eV^−1^) than Nita (η = 2.07 eV, S = 0.48 eV^−1^). A softer Mol is generally more polarizable and chemically reactive [22].

In terms of electrophilicity index (ω) which measures the stabilization energy when a system acquires additional electronic charge from its environment, both TIZ (7.31 eV) and Mol 7 (7.41 eV) have higher electrophilicity than Nita (6.60 eV), classifying them as strong electrophiles. This suggests a high capacity to participate in charge-transfer interactions, potentially leading to stronger binding with nucleophilic residues (e.g., serine, cysteine, histidine) in a protein active site [23].

#### 2.1.4. Molecular Electrostatic Potential (MEP) Analysis

**Figure 3:**
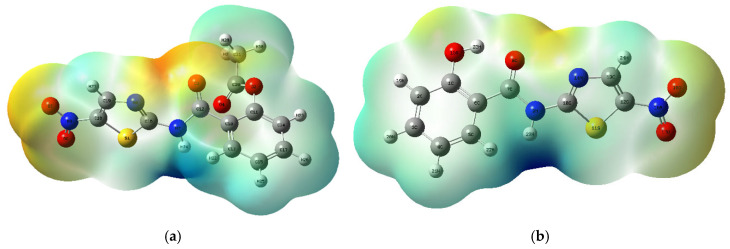

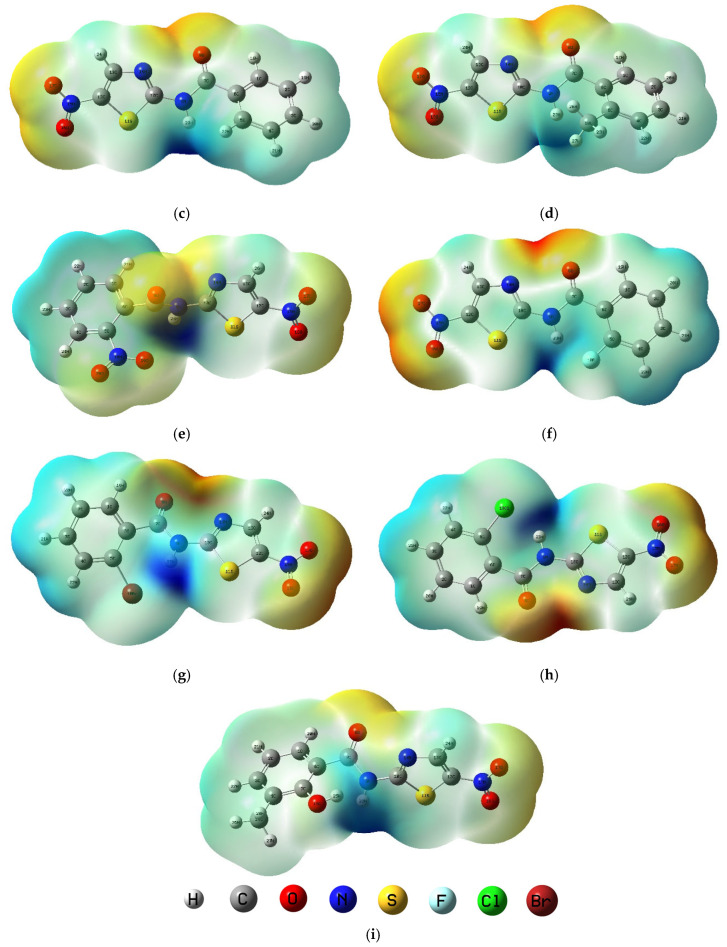
MEP maps of (**a**) Nita; (**b**) TIZ; (**c**) Mol 1; (**d**) Mol 2; (**e**) Mol 3; (**f**) Mol 4; (**g**) Mol 5; (**h**) Mol 6; (**i**) Mol 7.

The MEP maps (Figure 3) provide a visual representation of the charge distribution across the van der Waals surface, predicting sites for nucleophilic (red, negative potential) and electrophilic (blue, positive potential) attacks [24]. A consistent pattern is observed across all structures, showing that the negative (red) regions are localized over the oxygen atoms of the carbonyl and amide groups, identifying them as the prime hydrogen bond acceptors. On the other hand, the positive (blue) regions are found around the hydrogen atoms of the amide NH and, when present, the phenolic OH group (as in TIZ and Mol 7), marking them as strong hydrogen bond donors.

The MEP of TIZ and Mol 7 are particularly notable for their intense blue region over the phenolic -OH, providing a visual confirmation of their strong hydrogen bond donor capability, which is absent in Nita and most other analogues. This aligns perfectly with the high Max. ElPot values calculated in the QSAR analysis and underscores a critical pharmacophoric feature for target binding.

### 2.2. Quantitative Structure-Activity Relationship (QSAR) Analysis

A quantitative analysis of the molecular structure and properties of Nita, its active metabolite TIZ, and a series of systematically modified analogues (1–7) was conducted. The objective was to decode the impact of specific functional group substitutions on key physicochemical descriptors, thereby establishing a foundation for understanding their structure-activity relationships. The calculated molecular descriptors are summarized in Table 4.

#### 2.2.1. Molecular Size, Polarity, and the Prodrug-to-Drug Transformation

The accessible area (Acc. Area), a measure of molecular size, shows that the prodrug Nita is the largest Mol (212.93 Å^2^). Its metabolic conversion to TIZ results in a significant reduction in size (197.73 Å^2^), which is a common feature in prodrug design to enhance active species penetration [18]. The P-Area and Acc. P-Area are critical descriptors of molecular polarity and hydrophilicity, directly influencing solvation energy and passive diffusion [25,26]. Nita has the highest values (120.93 and 92.32 Å^2^, respectively), which drop considerably in TIZ (105.13 and 82.29 Å^2^, respectively), explaining the increased lipophilicity of the prodrug. Among the analogues, compound **3** (-NO_2_) is a clear outlier with dramatically high P-Area (141.46 Å^2^), indicating extreme polarity that would severely limit membrane permeability. Conversely, compound **7** (-OH, +CH_3_) exhibits the lowest polar surface areas, suggesting a structure optimized for traversing lipid bilayers.

#### 2.2.2. Electrostatic Profile and Implications for Molecular Recognition

The electrostatic potential (ESP) maps the molecular reactivity. The Min. ElPot identifies the most favorable site for electrophilic attack (e.g., hydrogen bonding with a protein donor). The halogenated compounds **4** (-F), **5** (-Br), and **6** (-Cl) display the most negative values (below −220 kJ/mol), characteristic of the strong electron-withdrawing nature and lone pairs of halogens, making them potent hydrogen bond acceptors. The Max. ElPot indicates the best site for nucleophilic interaction. TIZ shows the highest value (310.59 kJ/mol), which can be attributed to its free phenolic -OH group, a strong hydrogen bond donor. This is a key differentiator from Nita and most analogues (which lack this HBD), underscoring the importance of this group for target binding. The Min. LocIonPot, related to chemical reactivity, remains consistent across the series, indicating no major variations in stability from this perspective.

#### 2.2.3. Lipophilicity, Polarizability, and Hydrogen-Bonding Capacity

Log P is a paramount descriptor for predicting absorption and distribution. The data reveals a critical insight: the prodrug Nita is significantly more lipophilic (Log P = 2.17) than its active metabolite TIZ (Log P = −0.60). This is a classic prodrug strategy where a lipophilic moiety (OCOCH_3_) is added to enhance absorption, which is then cleaved metabolically to reveal the active, more hydrophilic drug (TIZ) [27]. Compound **2** (-CH_3_) is the most lipophilic analogue (Log P = 0.65), while compound **3** (-NO_2_) is the most hydrophilic (Log P = −1.96).

Polarizability, which influences van der Waals interactions and binding affinity [28], is highest for Nita and Mol 7, both of which contain bulkier substituents. The HBD/HBA counts further highlight the prodrug activation; the conversion from Nita (HBD = 0, HBA = 8) to TIZ (HBD = 1, HBA = 7) creates a critical hydrogen bond donor, which is often essential for high-affinity binding to a target protein.

#### 2.2.4. Integrated Analysis for Rational Drug Design

The systematic modifications to the Nita scaffold delineate clear structure–property–recognition relationships that can be exploited for rational design. Deacetylation to TIZ replaces the masked phenol with a free H-bond donor, sharpens the negative electrostatic basin around the salicylamide oxygen(s) [24], and trims steric bulk together stabilizing the conserved anchoring H-bond in PFOR while moderating lipophilicity and desolvation cost [29]. Within the analogue set, the strongly electron-withdrawing nitro group (Mol 3, –NO_2_) drives high polarity and a large polar surface area [30]; although this may be useful as a diagnostic probe for polar microenvironments, it is predicted to impair permeability and to hinder binding in the largely non-polar subpocket, consistent with weaker docking/QSAR scores. In contrast, halogenated analogues (Mols 4–6) increase polarizability and shape complementarity at modest log P, enabling halogen bonding (σ-hole interactions along C–X) to carbonyl or sulfur acceptors and π–halogen contacts rather than classical H-bond acceptance [14]; these features, together with improved membrane transit, make them well suited to occupy deeper hydrophobic grooves while preserving directional interactions. Mol 7 (–OH, +CH_3_) integrates the TIZ-like H-bond donor with a localized hydrophobe that strengthens dispersion within the non-polar pocket, lowers total polar surface relative to TIZ [31], and enhances softness/polarizability. Taken together, the QSAR/MEP trends converge on actionable rules: (i) retain the phenolic donor (or a prodrug mask that is efficiently unmasked) to secure the anchor; (ii) tune electron-withdrawing substitution to modulate surface electrostatics without exceeding polarity; (iii) leverage halogens for σ-hole/π interactions rather than putative H-bond acceptance [14]; (iv) add compact hydrophobes to enhance van der Waals complementarity without occluding the pocket. This integrated profile provides a robust basis for predictive modeling and prioritizes Mols 4 and 7, respectively, for maximal pocket engagement via polarizability/halogen bonding and a balanced permeability–binding trade-off for synthesis and experimental validation. 

### 2.3. Molecular Docking Studies: Binding Mode Analysis and Correlation with Physicochemical Properties

#### 2.3.1. Analysis of Docking Poses and Binding Affinities

**Figure 4:**
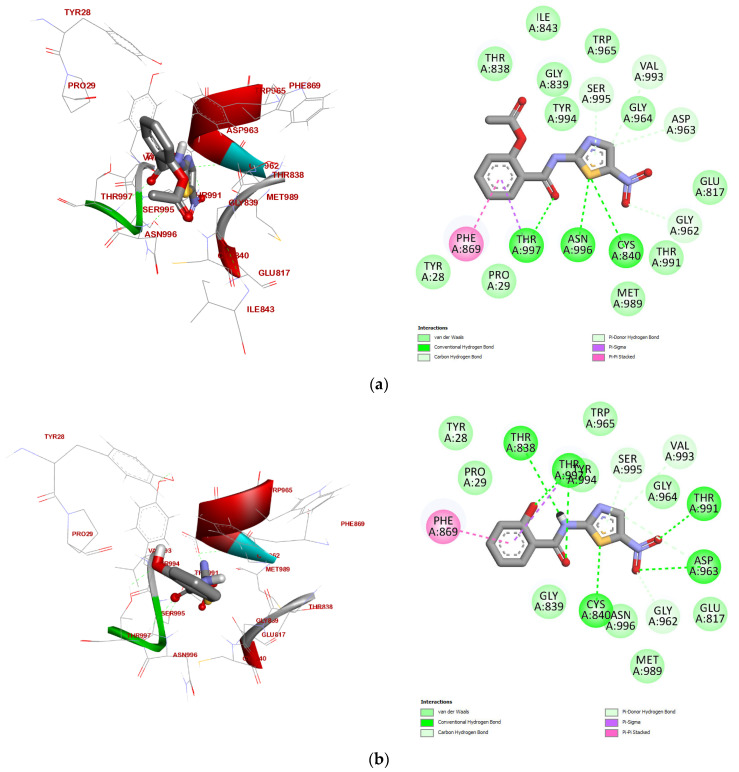

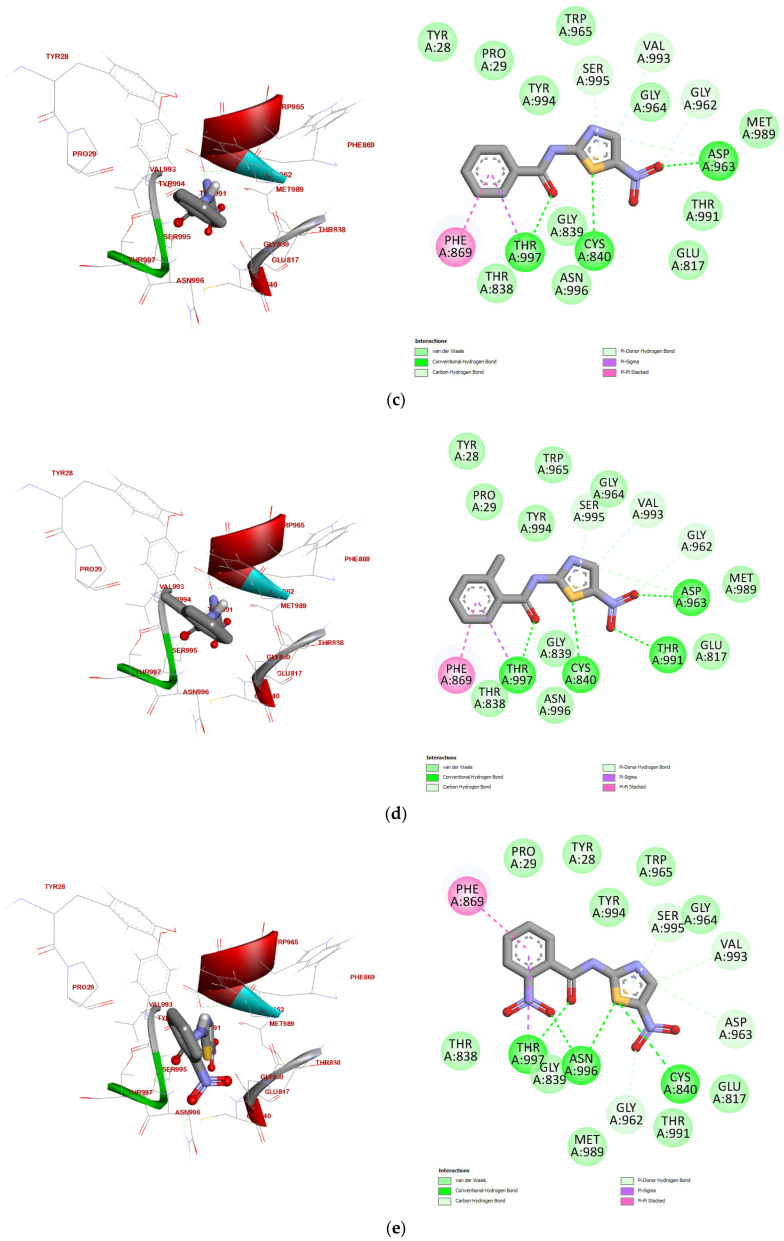

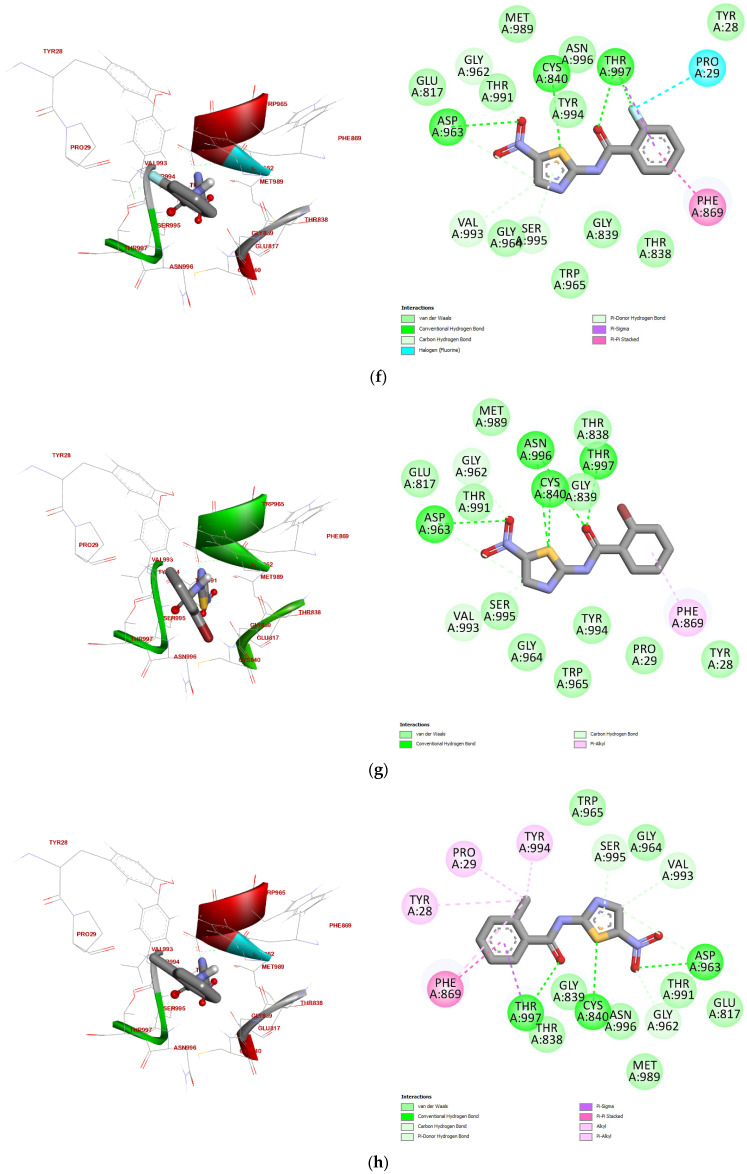

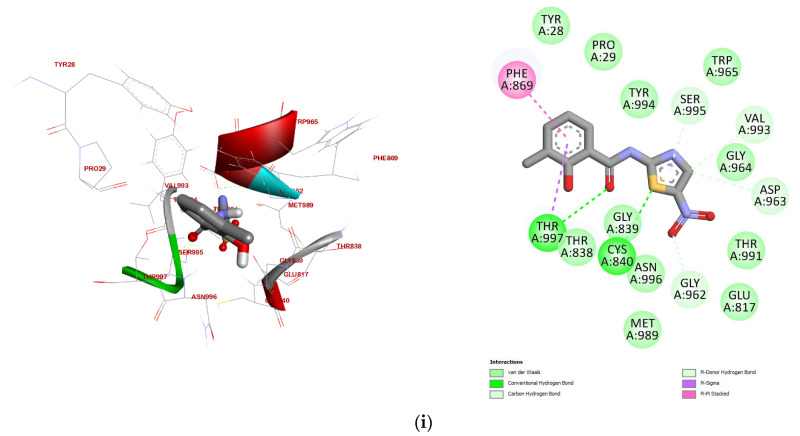
Three-dimensional view of ligand–PFOR interaction (left panel) and 2D view of the predicted types of interactions (right panel) for (**a**) Nita; (**b**) TIZ; (**c**) Mol 1; (**d**) Mol 2; (**e**) Mol 3; (**f**) Mol 4; (**g**) Mol 5; (**h**) Mol 6; (**i**) Mol 7.

From calculated binding affinities and interaction profiles for all compound in Figure 4 and presented in Table 5, Nita with binding affinity of −10.0 kcal/mol anchors the pocket with a triad of conventional H-bonds to Thr-996/Thr-997/Cys-840, reinforced by carbon/π-donor H-bonds to Gly-962/Asp-963/Val-993/Ser-995. A broad van der Waals belt (Tyr-28, Pro-29, Met-989, Thr-991, Glu-817, Gly-964, Trp-965, Tyr-994, Gly-839, Ile-843, Thr-838) stabilizes the pose, while π–π stacking with Phe-869 secures the aromatic core. This interaction pattern defines the benchmark. TIZ (binding affinity −9.6 kcal/mol) preserves Nita’s H-bonding logic but redistributes it across Thr-838/Thr-991/Thr-997/Cys-840/Asp-963, and it introduces a π–σ contact to Thr-997 alongside the conserved Phe-869 π–π stack. The network is slightly less cohesive than Nita’s, consistent with the small drop in affinity, yet it maintains all key recognition elements.

Mol 1 (−9.7 kcal/mol vs. Nita): Close to TIZ in strength, retains the canonical H-bond anchors (Thr-997/Cys-840/Asp-963) and the π–σ(Thr-997) + π–π(Phe-869) duo. Its extensive vdW set (Tyr-28, Pro-29, Trp-965, Tyr-994, Gly-964, Met-989, Thr-991, Glu-817, Asn-996, Gly-839, Thr-838) mirrors Nita’s, explaining the only modest loss in affinity.

Mol 2 (−10.0 kcal/mol vs. Nita): Equipotent with Nita, Mol 2 strengthens the hydrogen-bond scaffold (Thr-997/Cys-840/Asp-963/Thr-991) while keeping the secondary carbon/π-donor H-bonds (Gly-962/Asp-963/Val-993/Ser-995). The π–σ(Thr-997) and π–π(Phe-869) interactions are conserved, and the vdW contacts (Tyr-28, Pro-29, Trp-965, Tyr-994, Gly-964, Met-989, Glu-817, Asn-996, Gly-839, Thr-838) reproduce Nita’s hydrophobic envelope, hence the tie.

Mol 3 (−9.2 kcal/mol vs. Nita): This is the weakest binder in the set. Although it keeps the H-bond triad (Thr-997/Cys-840) and adds Asn-996, plus π–σ(Thr-997) and π–π(Phe-869), the overall contact density is reduced relative to Nita. The diminished cohesion of its vdW shell likely underlies the ~0.8 kcal·mol^−1^ penalty. Mol 4 (−10.1 vs. Nita). The top performer. It preserves Nita’s H-bond core (Thr-997/Cys-840/Asp-963) and broad vdW spread (Tyr-28, Thr-838, Gly-839, Trp-965, Gly-964, Glu-817, Thr-991, Met-989, Tyr-994, Asn-996), keeps π–σ(Thr-997) and π–π(Phe-869), and uniquely adds a halogen contact to Pro-29. This extra σ-hole interaction plausibly tightens orientation in the pocket, nudging affinity just beyond Nita. Mol 5 (−9.5 vs. Nita). With H-bonds to Thr-997/Cys-840/Asp-963/Asn-996 and a familiar secondary H-bond set, Mol 5 trades some stacking for π-alkyl contacts (notably to Phe-869). The shift toward alkyl/π-alkyl hydrophobics maintains recognition but explains the modest decline from Nita.

Mol 6 (−9.9 kcal/mol vs. Nita): Nearly equivalent to Nita. It combines the standard H-bond motif (Thr-997/Cys-840/Asp-963) and π–σ(Thr-997)/π–π(Phe-869) with an expanded hydrophobic lattice, alkyl/π-alkyl contacts across Phe-869, Tyr-28, Pro-29, Tyr-994—plus vdW to Thr-838/Gly-839/Asn-996/Met-989/Glu-817/Thr-991/Gly-964/Trp-965. The dense nonpolar packing likely compensates for minor electronic differences.

Mol 7 (−10.0 kcal/mol vs. Nita): Ties Nita despite a somewhat leaner H-bond set (Thr-997/Cys-840). The pose is propped up by the usual secondary H-bonds (Gly-962/Asp-963/Val-993/Ser-995), a comprehensive vdW network (Tyr-28, Pro-29, Trp-965, Tyr-994, Gly-964, Met-989, Thr-991, Glu-817, Asn-996, Gly-839, Thr-838), and conserved π–σ(Thr-997)/π–π(Phe-869) contacts. Affinity trend: M4 (−10.1) > Nita ≈ M2 ≈ M7 (−10.0) > M6 (−9.9) > M1 (−9.7) > TIZ (−9.6) > M5 (−9.5) > M3 (−9.2). Across the series, H-bonding to Thr-997/Cys-840 and π–π stacking with Phe-869 are the non-negotiables; π–σ to Thr-997 frequently appears as a booster. The halogen contact in Mol 4 is the only distinctive new interaction and coincides with the best score, supporting a halogen-enabled design as the clearest route to surpassing Nita.

#### 2.3.2. Conserved Binding Motif and the Role of Key Residues

Analysis of the docking poses reveals a well-defined and conserved binding motif within the PFOR active site, characterized by a core set of residues that engage the ligands through a combination of polar and hydrophobic interactions. This consistent binding mode provides a robust structural explanation for the high affinities observed across the series and highlights the complementary nature of the active site.

The binding is anchored by a highly conserved polar interaction network. Critically, all compounds form conventional hydrogen bonds with the side chains of Thr-997 and Cys-840. This result indicates that Cys-840 is a catalytically essential residue in PFOR that is, as reported elsewhere [32], involved in the covalent binding of the substrate’s thiamine pyrophosphate cofactor. Our finding that inhibitors consistently form a hydrogen bond with this residue provides a direct, structurally rationalized mechanism for competitive inhibition. Furthermore, the backbone or side chains of Asp-963 and Gly-962 consistently participate in carbon hydrogen bonds or act as π-donors, creating a stable, polar anchor for the ligands’ salicylamide core. A second, equally conserved element is the π- π-stacked interaction with Phe-869, which provides substantial stabilization through aromatic stacking. Notably, several key residues demonstrate their versatility by engaging in multiple interaction types. For instance, Thr-997 frequently forms not only a conventional H-bond but also a π-σ interaction, indicating close contact between its aliphatic side chain and an aromatic ring of the ligands. Similarly, Phe-869, in addition to π-stacking, sometimes engages in π-alkyl interactions (e.g., with Mol 5), further reinforcing its critical role in binding. Therefore, Thr-997 and Phe-869 also demonstrate as pivotal part of the active site architecture, providing a network of polar and hydrophobic interactions that stabilize the ligand pose. This conserved motif is also encapsulated by an extensive hydrophobic shell involving residues like Tyr-28, Pro-29, Met-989, and Tyr-994, which provide shape complementarity and van der Waals stabilization. The consistent and multifaceted interaction with Cys-840 is of particular mechanistic importance, suggesting these compounds act as competitive inhibitors by directly blocking this catalytically essential residue.

Deviations from this core motif provide valuable insights for structure-based design. Mol 4 (-F) is unique in forming a specific Halogen bond with Pro-29, an interaction distinct from the common hydrophobic contacts, which likely contributes to its superior binding affinity. The bulkier halogens in Mol 5 (-Br) and Mol 6 (-Cl) induce a shift in the interaction profile with Phe-869 from π-π stacking to π-alkyl and Alkyl interactions, reflecting their different steric and electronic properties. These subtle yet significant variations demonstrate how functionalization can fine-tune the binding mode, providing a clear rationale for the differences in computed affinity and highlighting specific residues that can be targeted for further optimization. Despite these peripheral variations, the high degree of consistency in the core binding mode, particularly the conserved interactions with Thr-997, Cys-840, and Phe-869, underscores the reliability of the docking protocol and the robustness of the identified conserved motif.

#### 2.3.3. Prodrug vs. Active Metabolite: Nita and TIZ

The docking results provide a structural rationale for the binding of the prodrug Nita and its active metabolite TIZ. Nita demonstrated a strong binding affinity of −10.0 kcal/mol, stabilized by the conserved interactions with Thr-997, Cys-840, and Phe-869, alongside an extensive network of van der Waals contacts. TIZ showed a slightly weaker affinity (−9.6 kcal/mol) but engaged in a more extensive hydrogen bond network, forming conventional H-bonds with Thr-991 and Asp-963 in addition to Thr-997 and Cys-840. This is likely facilitated by its free phenolic hydroxyl group, which aligns with the QSAR analysis that identified TIZ’s high Max. ElPot (a region of high electrophilicity) as a key feature. The slight difference in affinity suggests that while TIZ’s additional H-bonds are favorable, the bulkier, more lipophilic acetoxy group of Nita provides superior van der Waals stabilization within the hydrophobic subpocket of PFOR, compensating for its reduced hydrogen-bonding capacity.

#### 2.3.4. Correlation Between QSAR Descriptors and Docking Performance

A strong correlation exists between the physicochemical properties from the QSAR analysis and the observed docking results. The halogens introduced in Compounds **4**, **5**, and **6** generally increased lipophilicity (Log P of −0.06, 0.34, and −0.43, respectively) compared to TIZ (−0.60). This enhanced hydrophobicity correlates with their strong binding affinities (−10.1, −9.5, and −9.9 kcal/mol), particularly for Compound **4** (-F), which achieved the highest affinity. Its optimal balance is evidenced by a specific Halogen bond with Pro-29, a unique interaction not seen with other halogens, demonstrating how a small, electronegative atom can confer a distinct advantage.

The QSAR analysis predicted that halogenated compounds would be strong hydrogen bond acceptors due to their highly negative Min. ElPot values. The docking results confirm their ability to maintain key H-bonds, particularly with Thr-997 and Cys-840. Conversely, Compound **3** (-NO_2_), with its exceptionally high polarity (P-Area = 141.46 Å^2^) and hydrophilic character (Log P = −1.96), showed the weakest binding (−9.2 kcal/mol). Its binding pose is notably disrupted, as it fails to engage effectively with the key hydrophobic residues that form the conserved shell around other ligands. Instead of forming robust hydrophobic contacts, it relies more heavily on polar interactions, which is insufficient for high-affinity binding in this pocket.

Compound **7**, which incorporates the HBD phenolic -OH of TIZ and an additional methyl group, successfully achieves a high binding affinity (−10.0 kcal/mol). This aligns perfectly with the QSAR data, which highlighted its combination of HBD capacity and a relatively low polar surface area, making it a well-balanced candidate for both binding and permeability.

## 3. Materials and Methods

### 3.1. Molecular Modeling

The initial molecular structures of Nita, TIZ, and all designed analogues were constructed using GaussView 5.0 software [33]. The analogues were systematically derived from the Nita scaffold by replacing the native OCOCH_3_ group with the following substituents: Mol 1 (-H), Mol 2 (-CH_3_), Mol 3 (-NO_2_), Mol 4 (-F), Mol 5 (-Br), Mol 6 (-Cl), and Mol 7 (-OH, with an additional -CH_3_ group on the adjacent carbon of the aromatic ring). Subsequent computational modeling was performed to determine the precise three-dimensional geometries and electronic properties. All calculations were conducted using Density Functional Theory (DFT) as implemented in the Gaussian 09 software package [34]. The geometries of all compounds were fully optimized without any symmetry constraints. These optimizations employed the hybrid B3LYP functional (Becke’s three-parameter exchange functional and the Lee-Yang-Parr correlation functional) in conjunction with the 6-311+G(d,p) basis set, the absence of negative frequencies in the calculated spectrum suggests the stability of the optimized molecular structure, indicating the suitability of the model for the studied analogues. This outcome affirms that the IR spectrum in Supplementary Files was derived from an optimized structure, underscoring the accuracy and reliability of the method. This level of theory provides a reliable description of molecular structures and electronic properties for organic systems [35]. Frequency calculations were performed on the optimized structures at the same level of theory to confirm the absence of imaginary frequencies, thereby verifying that each structure represented a true minimum on the potential energy surface. The optimized geometries were used for subsequent calculation of molecular electrostatic potentials and frontier molecular orbitals.

### 3.2. Quantitative Structure-Activity Relationship

Following the DFT-based geometry optimization, a quantitative structure-activity relationship (QSAR) analysis was performed using the SCIGRESS 3.0 software package [24,36]. A targeted set of molecular descriptors was computed from the DFT-optimized structures to encode key structural and electronic properties. These included geometrical descriptors such as Accessible Area, Polar Area (P-Area), and Accessible Polar Area (Acc. P-Area), which quantify molecular size and polarity. Electronic properties were characterized by electrostatic potential extrema (Min. ElPot, Max. ElPot) and the Minimum Local Ionization Potential (Min. LocIonPot). Key physicochemical properties were also calculated, namely the octanol-water partition coefficient (Log P) and the counts of hydrogen bond donors (HBD) and acceptors (HBA). The use of DFT-optimized structures ensured consistent and accurate calculation of electronic descriptors.

### 3.3. Molecular Docking

Molecular docking simulations were performed to predict the binding modes and affinities of Nita, TIZ, and their analogues (1–7) within the active site of the PFOR enzyme. The three-dimensional structure of the PFOR enzyme was retrieved from protein databank (PDB ID: 1kek) [37] and prepared for docking using AutoDock Tools 4 [38]. This preparation included the addition of polar hydrogen atoms, merging of non-polar hydrogens, and assignment of Gasteiger charges to ensure accurate electrostatic representation. The ligand structures were similarly prepared, and their torsional bonds were defined. Docking calculations were carried out using AutoDock Vina [39]. The search space was defined as a grid box encompassing the known active site of PFOR. The grid box was centered at coordinates (center_x = −22.458, center_y = −56.459, center_z = 29.352) with dimensions of 40 Å × 40 Å × 40 Å, providing sufficient space for comprehensive ligand sampling and rotation. For each ligand, the best pose was selected based on the calculated binding affinity (kcal/mol) and the consistency of the binding mode. The resulting protein-ligand complexes were visualized and analyzed using Biovia Discovery Studio 2021 to delineate specific molecular interactions.

## 4. Conclusions

Multi-scale analysis (DFT → QSAR → docking) resolves a coherent recognition code for thiazolides at PFOR and converts it into testable design rules. Across the series, productive binding is gated by a conserved hydrogen-bond dyad to Thr-997/Cys-840 and a π–π stack with Phe-869, frequently reinforced by a π–σ contact to Thr-997: loss or weakening of any of these motifs predicts an immediate penalty in affinity. Deacetylation of nitazoxanide to tizoxanide unmasks the phenolic donor and increases local electrophilicity, yet its pose is marginally less cohesive than nitazoxanide because of subtle changes in pocket packing (TIZ −9.6 vs. Nita −10.0 kcal mol^−1^). By contrast, strategic halogenation adds a σ-hole interaction that locks orientation without disrupting the donor network; Mol 4 exhibits the best score (−10.1), while Mols 2 and 7 match the reference (−10.0) by preserving the full contact triad and hydrophobic belt. These results nominate halogen-enabled scaffolds that retain the phenolic H-bond donor and enforce the Thr-997/Cys-840/Phe-869 triad as the most credible route to exceed nitazoxanide, and they yield concrete, falsifiable predictions for synthesis and assay (enzyme kinetics and pocket-resolved biophysics). Prospective validation with explicit-solvent free-energy calculations and microsecond MD, followed by enzymology and whole-cell tests, should rapidly discriminate the highest-value analogues for translation.

## Figures and Tables

**Table 1:** B3LYP/6-311+G(d,p) calculated dipole moment in Debye (D) of Nita, TIZ and 7 analogues.

Structure	Dipole Moment (D)
Nita	7.508
TIZ	6.389
Mol 1	7.667
Mol 2	7.356
Mol 3	9.211
Mol 4	8.367
Mol 5	8.037
Mol 6	8.085
Mol 7	6.159

**Table 2:** B3LYP/6-311+G(d,p) calculated frontier molecular orbitals energies and band gap of Nita, TIZ and 7 analogues.

Structure	LUMO (eV)	HOMO (eV)	ΔE (eV)
Nita	−3.159	−7.309	4.1491943
TIZ	−3.342	−6.995	3.6523141
Mol 1	−3.205	−7.326	4.1206223
Mol 2	−3.201	−7.327	4.1260646
Mol 3	−3.393	−7.511	4.117629
Mol 4	−3.191	−7.257	4.0653832
Mol 5	−3.188	−7.274	4.0860638
Mol 6	−3.196	−7.281	4.0852475
Mol 7	−3.358	−6.932	3.5742174

**Table 3:** B3LYP/6-311+G(d,p) calculated global reactivity descriptors of Nita, TIZ and 7 analogues.

Structure	IP (eV)	EA (eV)	μ (eV)	η (eV)	S (eV^−1^)	ω (eV)	χ
Nita	7.309	−3.159	−5.234	2.074	0.482	6.603	5.234
TIZ	6.995	−3.342	−5.168	1.826	0.547	7.314	5.168
Mol 1	7.326	−3.205	−5.265	2.060	0.485	6.728	5.265
Mol 2	7.327	−3.201	−5.264	2.063	0.485	6.717	5.264
Mol 3	7.511	−3.393	−5.452	2.059	0.486	7.218	5.452
Mol 4	7.257	−3.191	−5.224	2.033	0.492	6.713	5.224
Mol 5	7.274	−3.188	−5.231	2.043	0.489	6.698	5.231
Mol 6	7.281	−3.196	−5.238	2.043	0.489	6.717	5.238
Mol 7	6.932	−3.358	−5.145	1.787	0.559	7.406	5.145

**Table 4:** Calculated molecular descriptors for Nita, TIZ, and functionalized analogues.

QSAR Descriptor	Nita	TIZ	1	2	3	4	5	6	7
Acc. Area (Å^2^)	212.93	197.73	191.82	197.20	198.80	194.98	202.89	201.02	205.15
P-Area (Å^2^)	120.93	105.13	107.28	112.02	141.46	103.49	104.98	103.96	98.34
Acc. P-Area (Å^2^)	92.32	82.29	81.48	82.84	110.23	77.14	78.36	77.77	74.27
Min. ElPot. (kJ/mol)	−196.79	−179.58	−205.05	−206.49	−186.33	−223.47	−220.39	−220.60	−157.24
Max. ElPot. (kJ/mol)	278.07	310.59	294.76	297.24	307.18	249.72	251.53	253.34	260.50
Min. LocIonPot (kJ/mol)	39.18	39.95	39.36	39.44	39.86	39.75	39.88	39.81	39.86
Polarizability	62.02	58.73	58.17	59.64	59.89	58.51	59.65	59.27	60.32
Log P	2.17	−0.60	0.48	0.65	−1.96	−0.06	0.61	0.34	−0.43
HBD	0	1	0	0	0	0	0	0	1
HBA	8	7	6	6	9	6	6	6	7

**Table 5:** Docking Results and Key Interactions for Nita, TIZ, and Analogues with PFOR.

Compound	Binding Affinity (kcal/mol)	Key Interactions with PFOR
Nita	−10.0	Conventional H-bond (Thr-996, Thr-997, Cys-840), Carbon H-bond and π-donor H-bond (Gly-962, Asp-963, Val-993, Ser-995), vdW (Tyr-28, Pro-29, Met-989, Thr-991, Glu-817, Gly-964, Trp-965, Tyr-994, Gly-839, Ile-843, Thr838), π- π-stacked (Phe-869)
TIZ	−9.6	Conventional H-bond (Thr-838, Thr-997, Cys-840, Thr-991, Thr-838, Asp-963), Carbon H-bond and π-donor H-bond (Gly-962, Asp-963, Val-993, Ser-995), vdW (Tyr-28, Pro-29, Trp-965, Gly-964, Glu-817, Asn-996, Met-989, Gly-839), π-σ (Thr-997), π-π-stacked (Phe-869)
Molecule 1	−9.7	Conventional H-bond (Thr-997, Cys-840, Asp-963), Carbon H-bond and π-donor H-bond (Gly-962, Asp-963, Val-993, Ser-995), vdW (Tyr-28, Pro-29, Trp-965, Tyr-994, Gly-964, Met-989, Thr-991, Glu-817, Asn-996, Gly-839, Thr-838), π-σ (Thr-997), π-π-stacked (Phe-869)
Molecule 2	−10.0	Conventional H-bond (Thr-997, Cys-840, Asp-963, Thr-991), Carbon H-bond and π-donor H-bond (Gly-962, Asp-963, Val-993, Ser-995), vdW (Tyr-28, Pro-29, Trp-965, Tyr-994, Gly-964, Met-989, Glu-817, Asn-996, Gly-839, Thr-838), π-σ (Thr-997), π-π-stacked (Phe-869)
Molecule 3	−9.2	Conventional H-bond (Thr-997, Cys-840, Asn-996), Carbon H-bond and π-donor H-bond (Gly-962, Asp-963, Val-993, Ser-995), vdW (Tyr-28, Pro-29, Trp-965, Tyr-994, Gly-964, Met-989, Thr-991, Glu-817, Gly-839, Thr-838), π-σ (Thr-997), π-π-stacked (Phe-869)
Molecule 4	−10.1	Conventional H-bond (Thr-997, Cys-840, Asp-963), Carbon H-bond and π-donor H-bond (Gly-962, Asp-963, Val-993, Ser-995), vdW (Tyr-28, Thr-838, Gly-839, Trp-965, Gly-964, Glu-817, Thr-991, Met-989, Tyr-994, Asn-996), Halogen (Pro-29), π-σ (Thr-997), π-π-stacked (Phe-869)
Molecule 5	−9.5	Conventional H-bond (Thr-997, Cys-840, Asp-963, Asn-996), Carbon H-bond (Gly-962, Asp-963, Val-993, Ser-995), vdW (Tyr-28, Pro-29, Trp-965, Tyr-994, Gly-964, Ser-995, Met-989, Thr-991, Glu-817, Gly-839, Thr-838), π-alkyl (Phe-869)
Molecule 6	−9.9	Conventional H-bond (Thr-997, Cys-840, Asp-963), Carbon H-bond and π-donor H-bond (Gly-962, Asp-963, Val-993, Ser-995), vdW (Thr-838, Gly-839, Asn-996, Met-989, Glu-817, Thr-991, Gly-964, Trp-965), π-σ (Thr-997), π-π-stacked (Phe-869), Alkyl and π-alkyl (Phe-869, Tyr-28, Pro-29, Tyr-994)
Molecule 7	−10.0	Conventional H-bond (Thr-997, Cys-840), Carbon H-bond and π-donor H-bond (Gly-962, Asp-963, Val-993, Ser-995), vdW (Tyr-28, Pro-29, Trp-965, Tyr-994, Gly-964, Met-989, Thr-991, Glu-817, Asn-996, Gly-839, Thr-838), π-σ (Thr-997), π-π-stacked (Phe-869)

## Data Availability

The original contributions presented in this study are included in the article/Supplementary Materials. Further inquiries can be directed to the corresponding authors.

## Supplementary Materials

The following supporting information can be downloaded at: https://www.mdpi.com/article/10.3390/ijms262311578/s1.
