# Supplementary figures and images for: First-Principles Modeling of Nitazoxanide Analogues as Prospective PFOR-Targeted Antibacterials
Source: Int J Mol Sci. 2025 Nov 28;26(23):11578. doi: 10.3390/ijms262311578 (PMC12691758; doi:10.3390/ijms262311578)

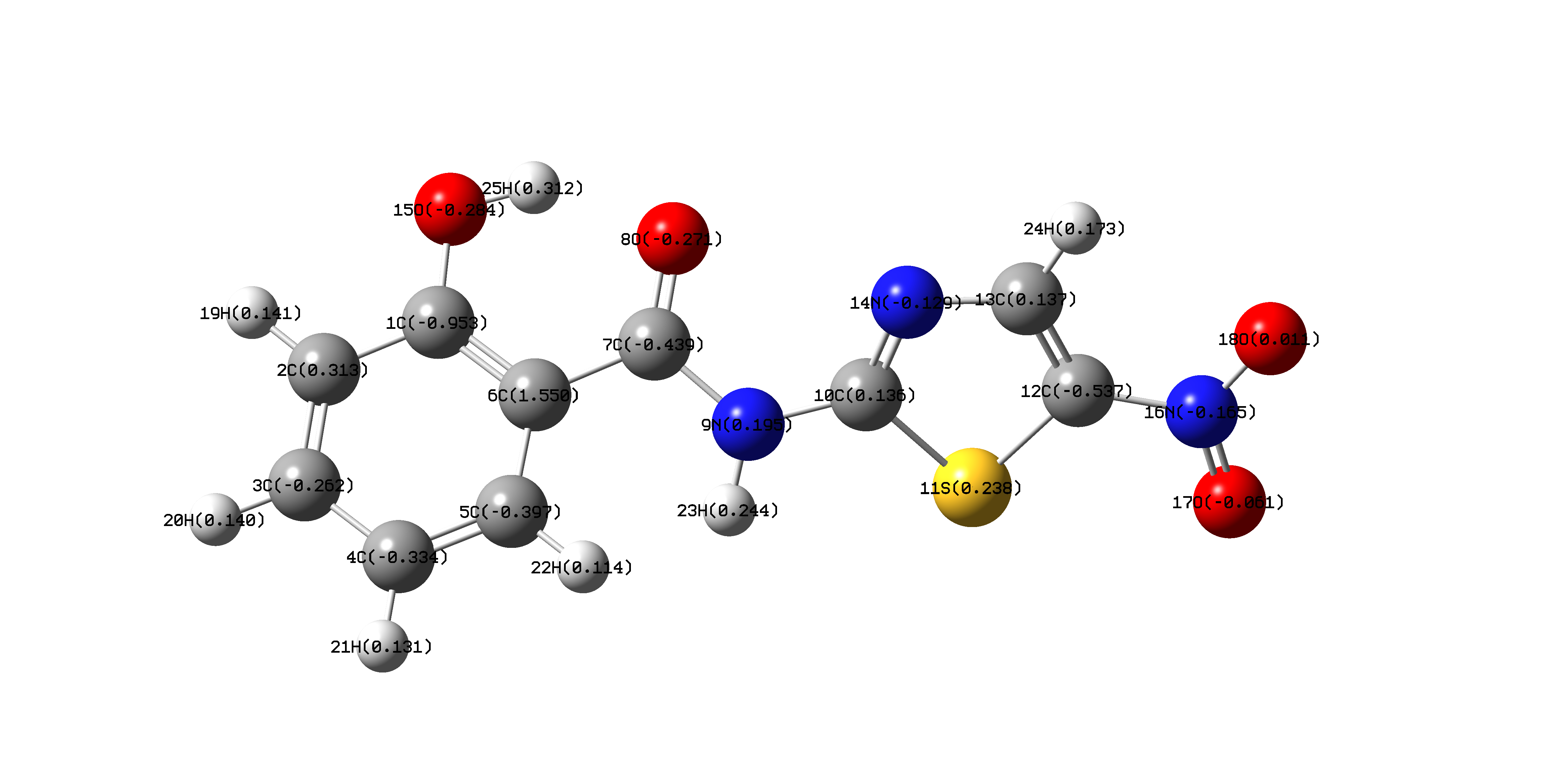

Supplement: Supplementary file 1 [file ijms-26-11578-s001.zip › TIZ/CHARGE DISTRIBUTION 1.png]

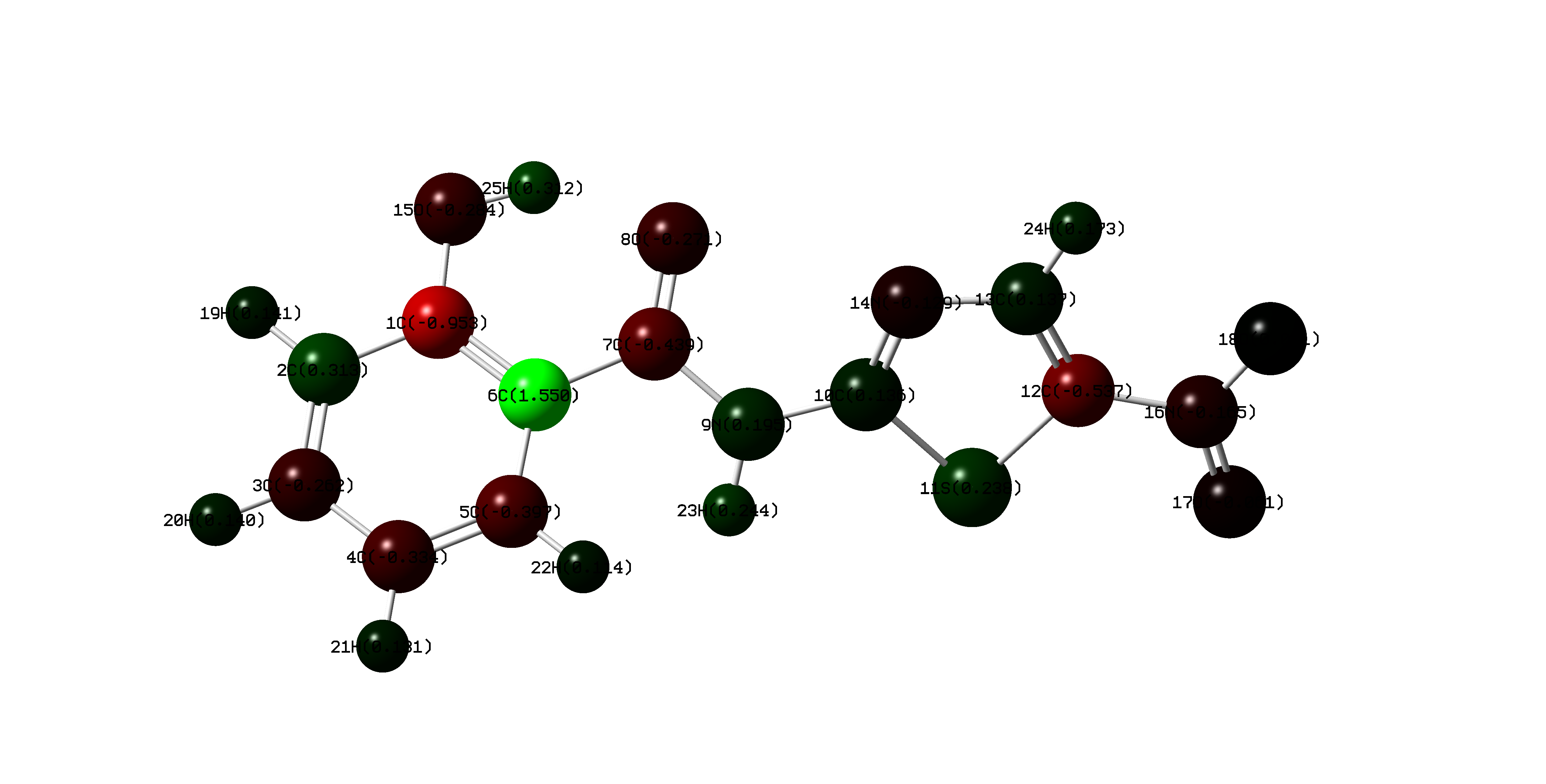

Supplement: Supplementary file 1 [file ijms-26-11578-s001.zip › TIZ/CHARGE DISTRIBUTION 2.png]

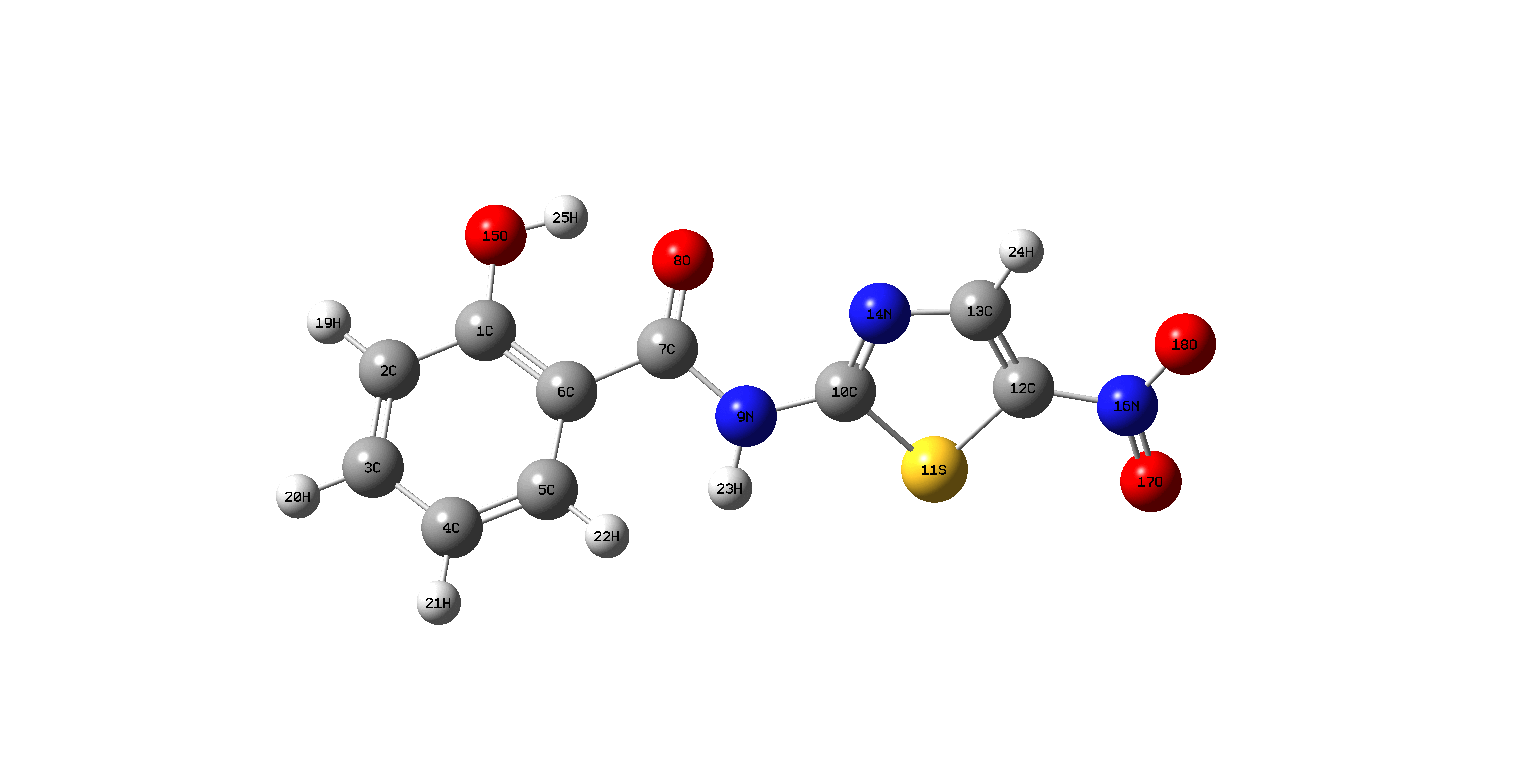

Supplement: Supplementary file 1 [file ijms-26-11578-s001.zip › TIZ/FREQ MOVIES.gif]

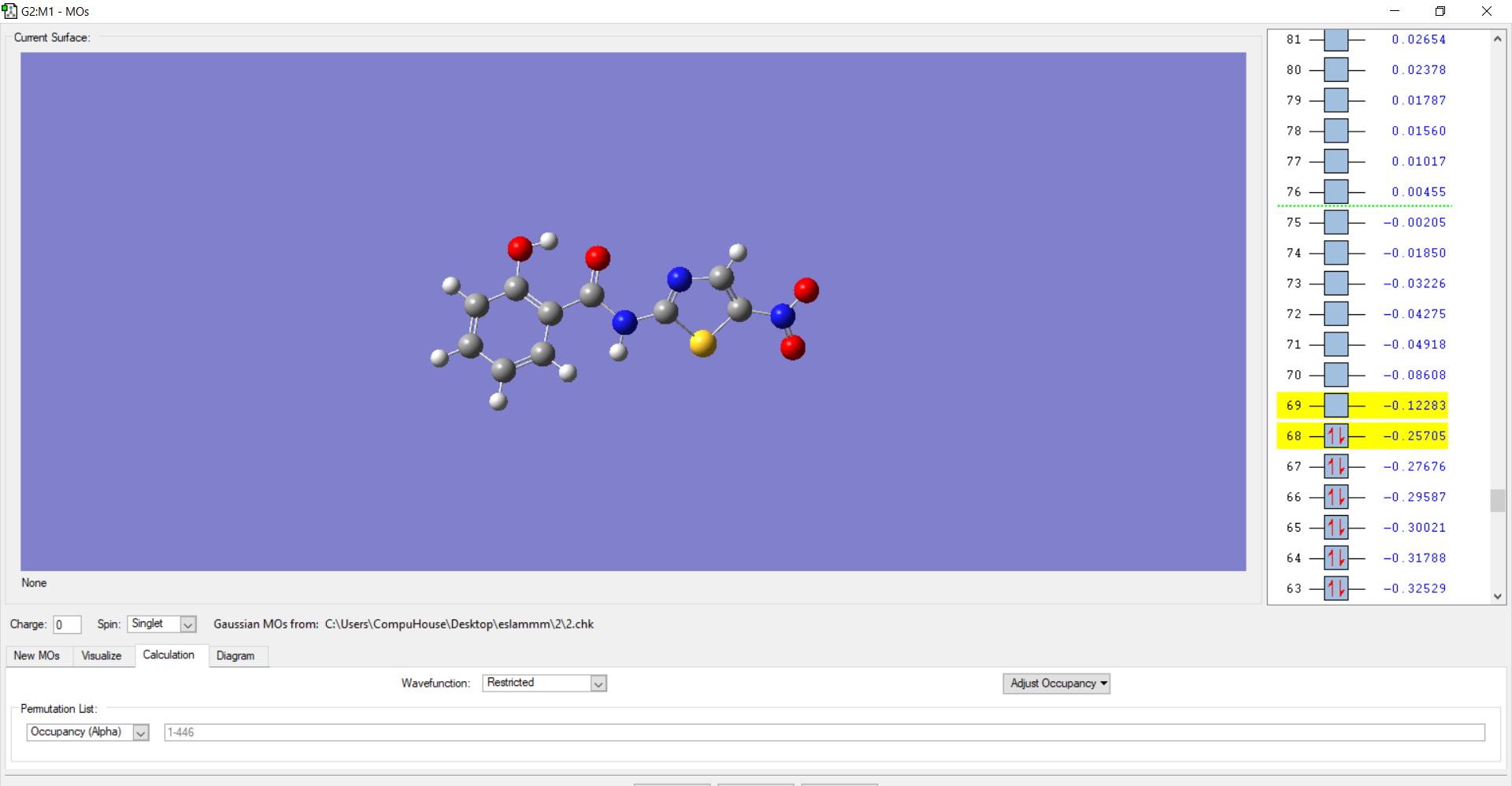

Supplement: Supplementary file 1 [file ijms-26-11578-s001.zip › TIZ/HOMO LUMO.JPG]

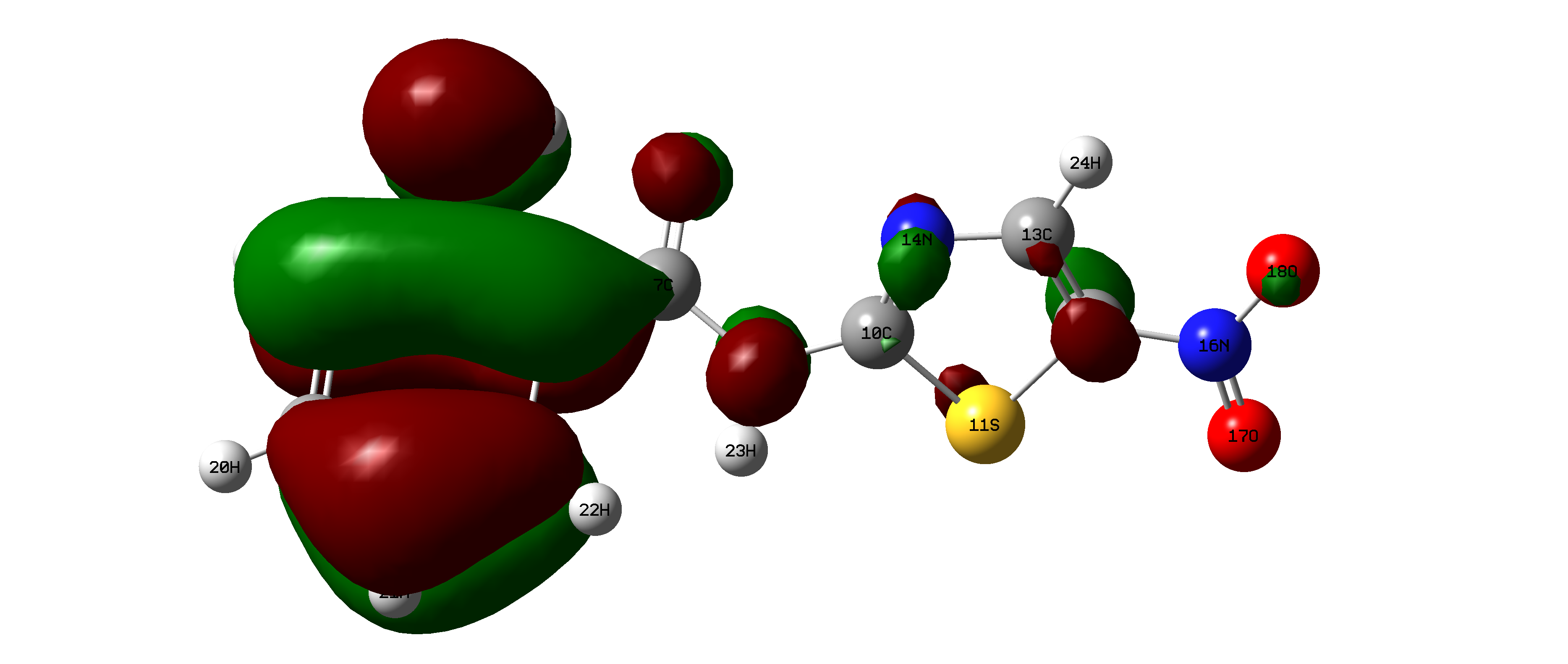

Supplement: Supplementary file 1 [file ijms-26-11578-s001.zip › TIZ/HOMO.png]

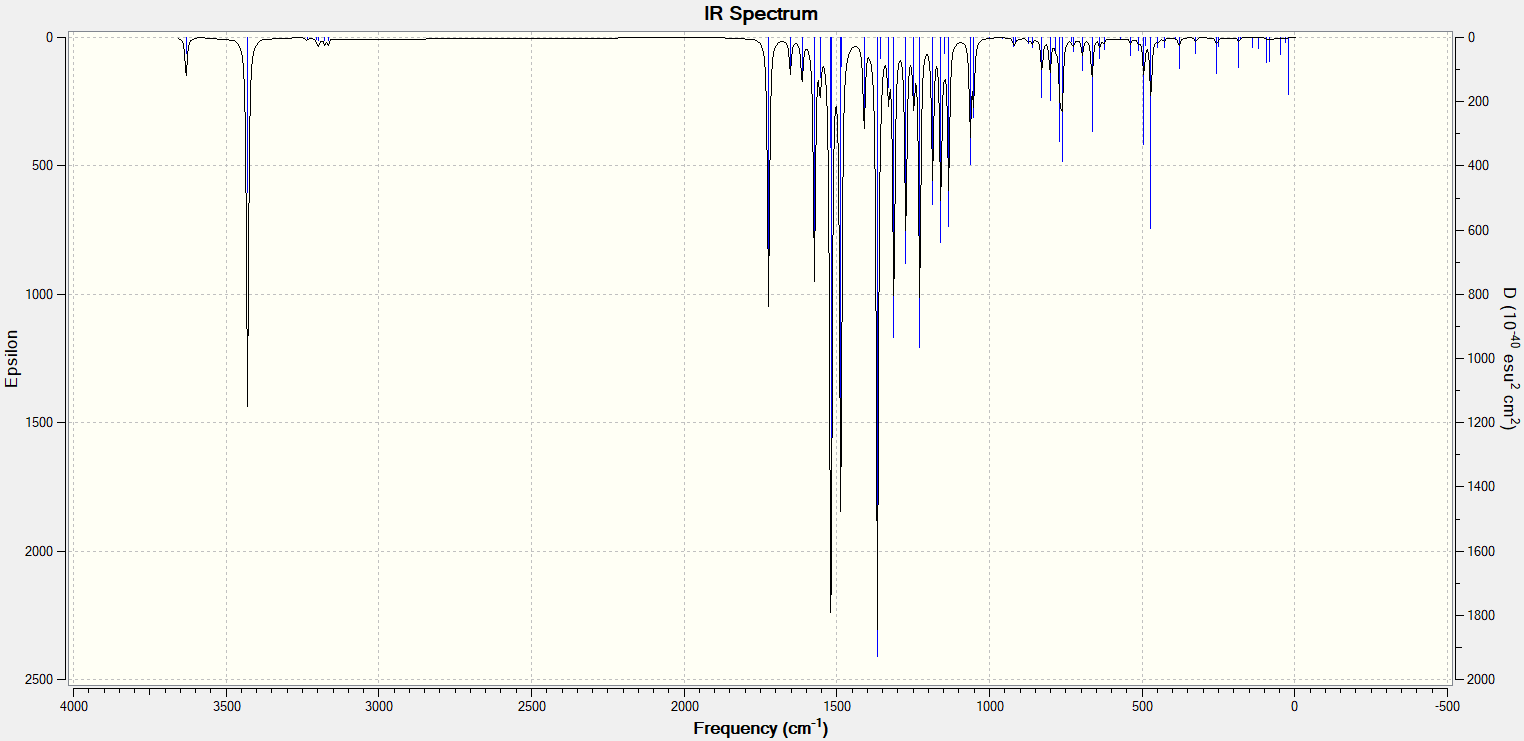

Supplement: Supplementary file 1 [file ijms-26-11578-s001.zip › TIZ/IR 2.png]

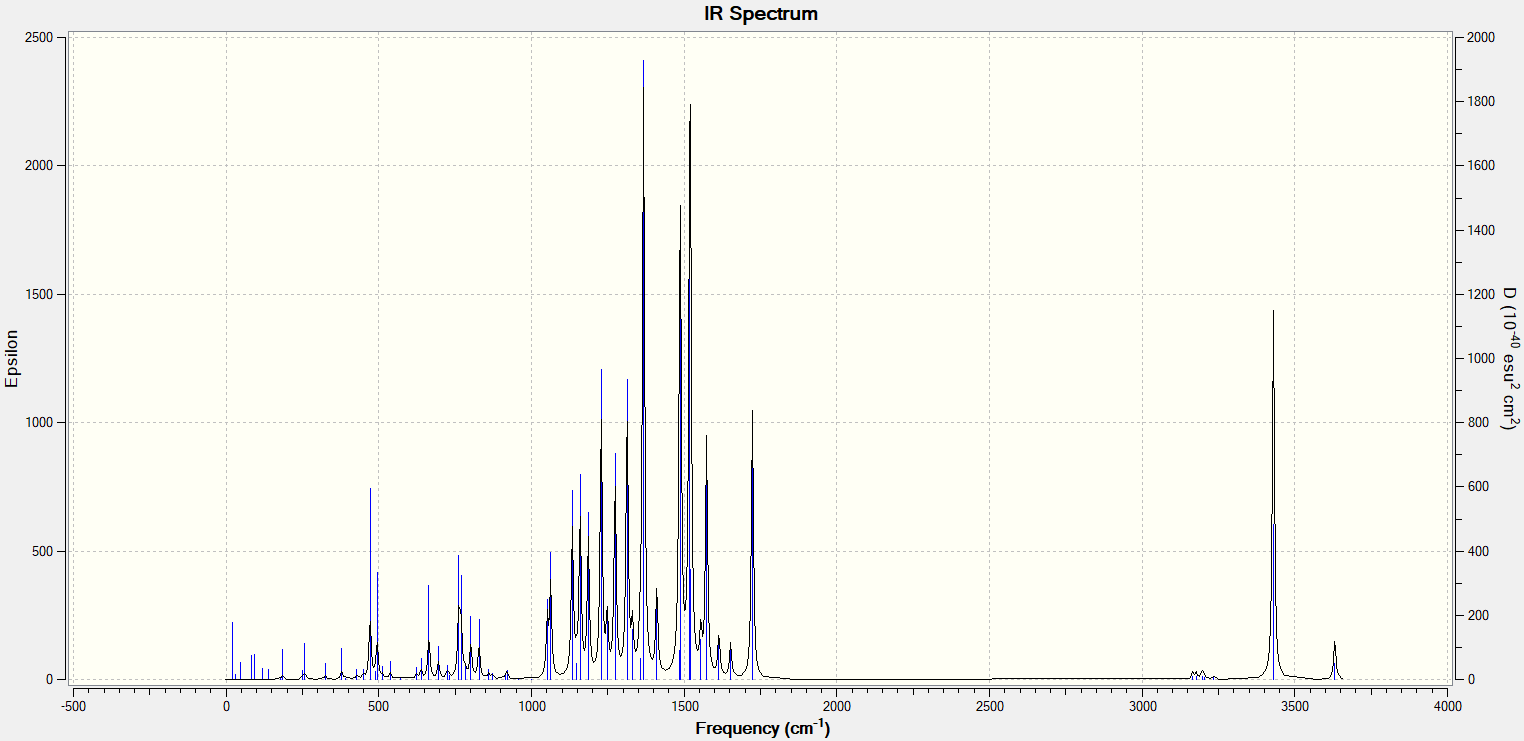

Supplement: Supplementary file 1 [file ijms-26-11578-s001.zip › TIZ/IR.png]

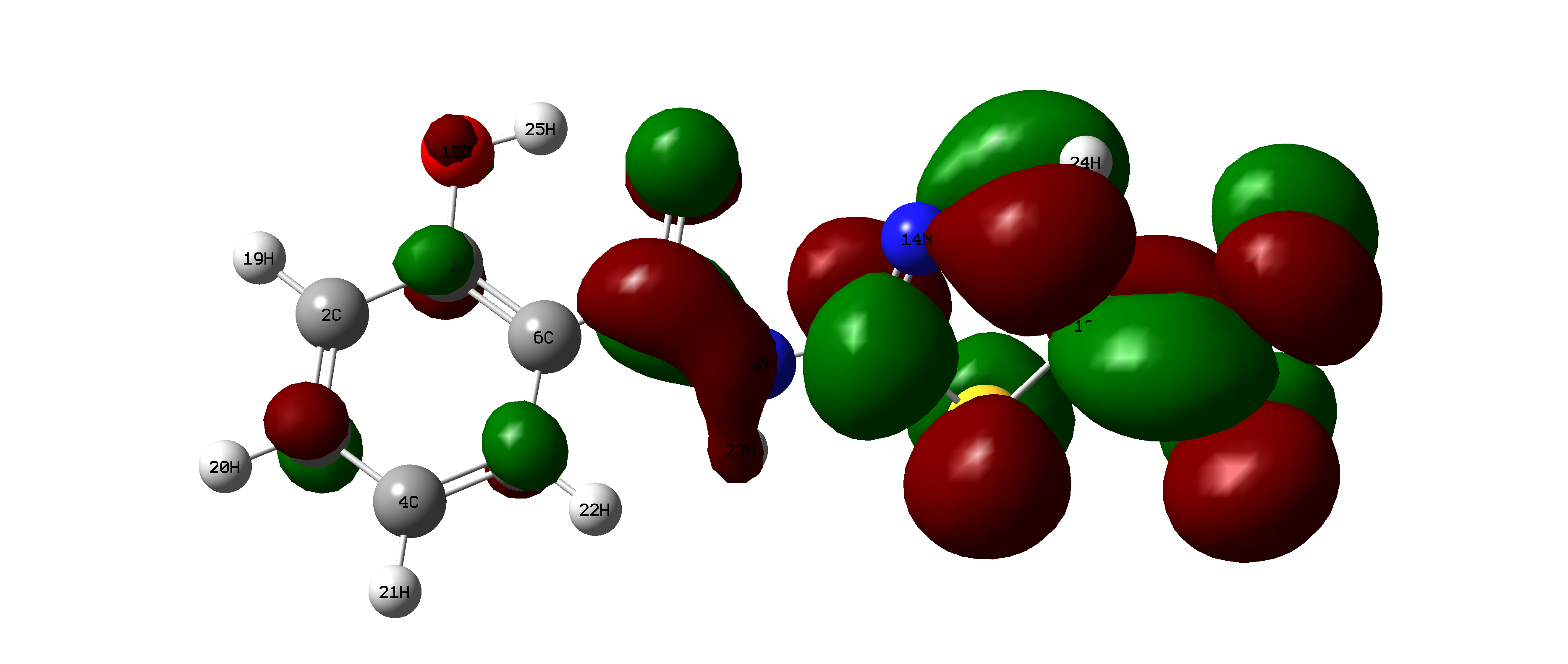

Supplement: Supplementary file 1 [file ijms-26-11578-s001.zip › TIZ/LUMO.png]

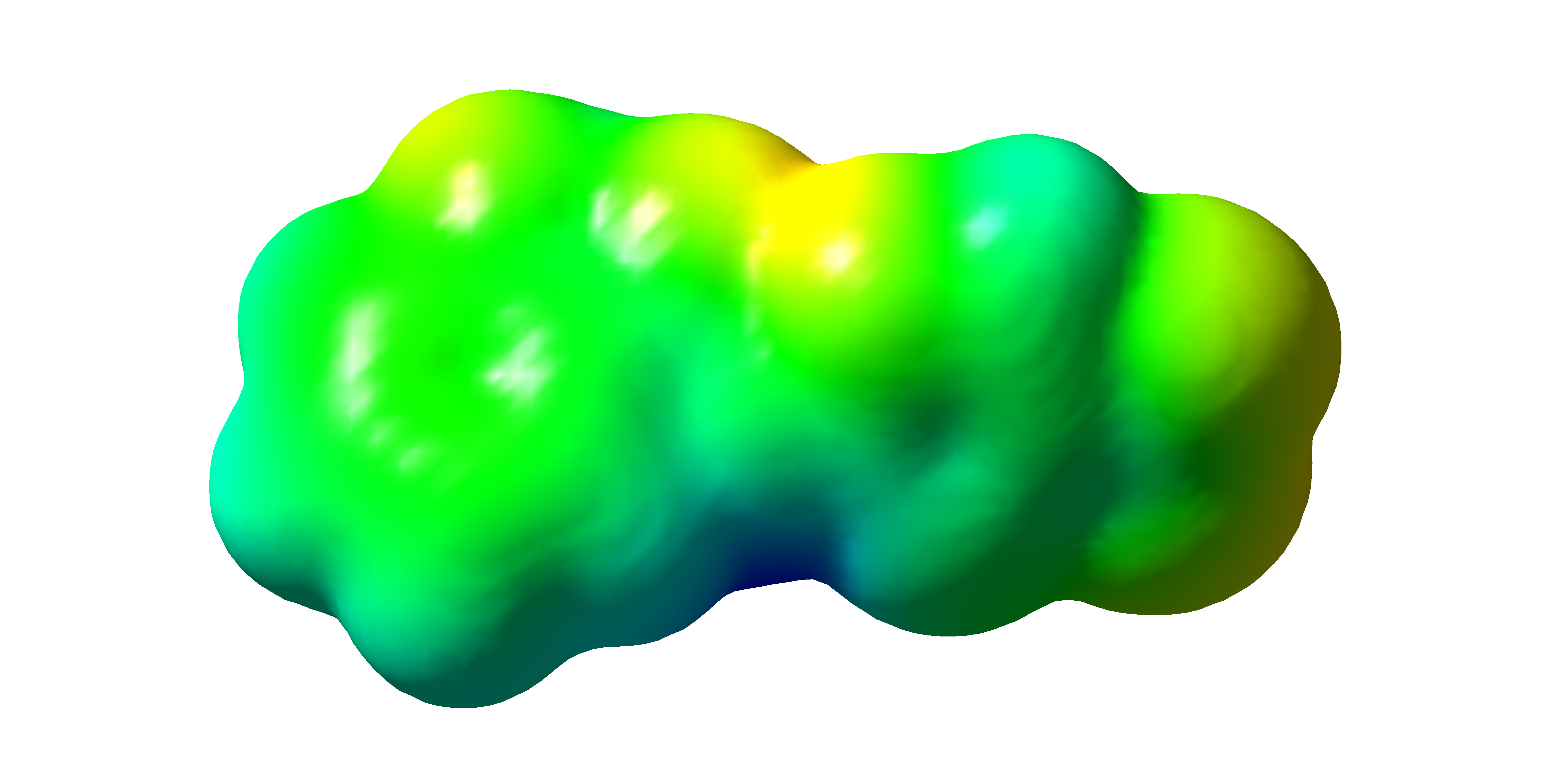

Supplement: Supplementary file 1 [file ijms-26-11578-s001.zip › TIZ/MEP1.png]

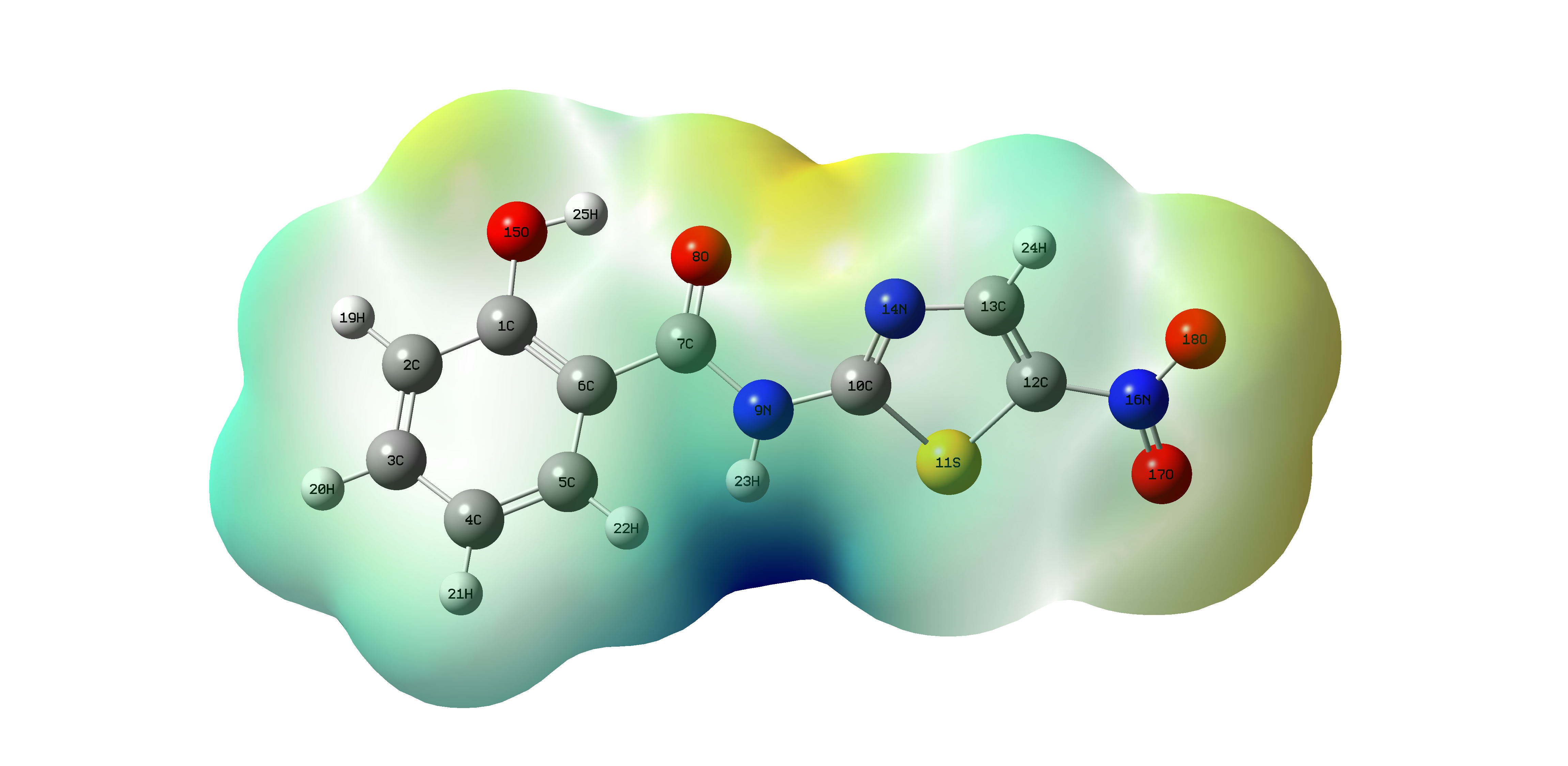

Supplement: Supplementary file 1 [file ijms-26-11578-s001.zip › TIZ/MEP2.png]

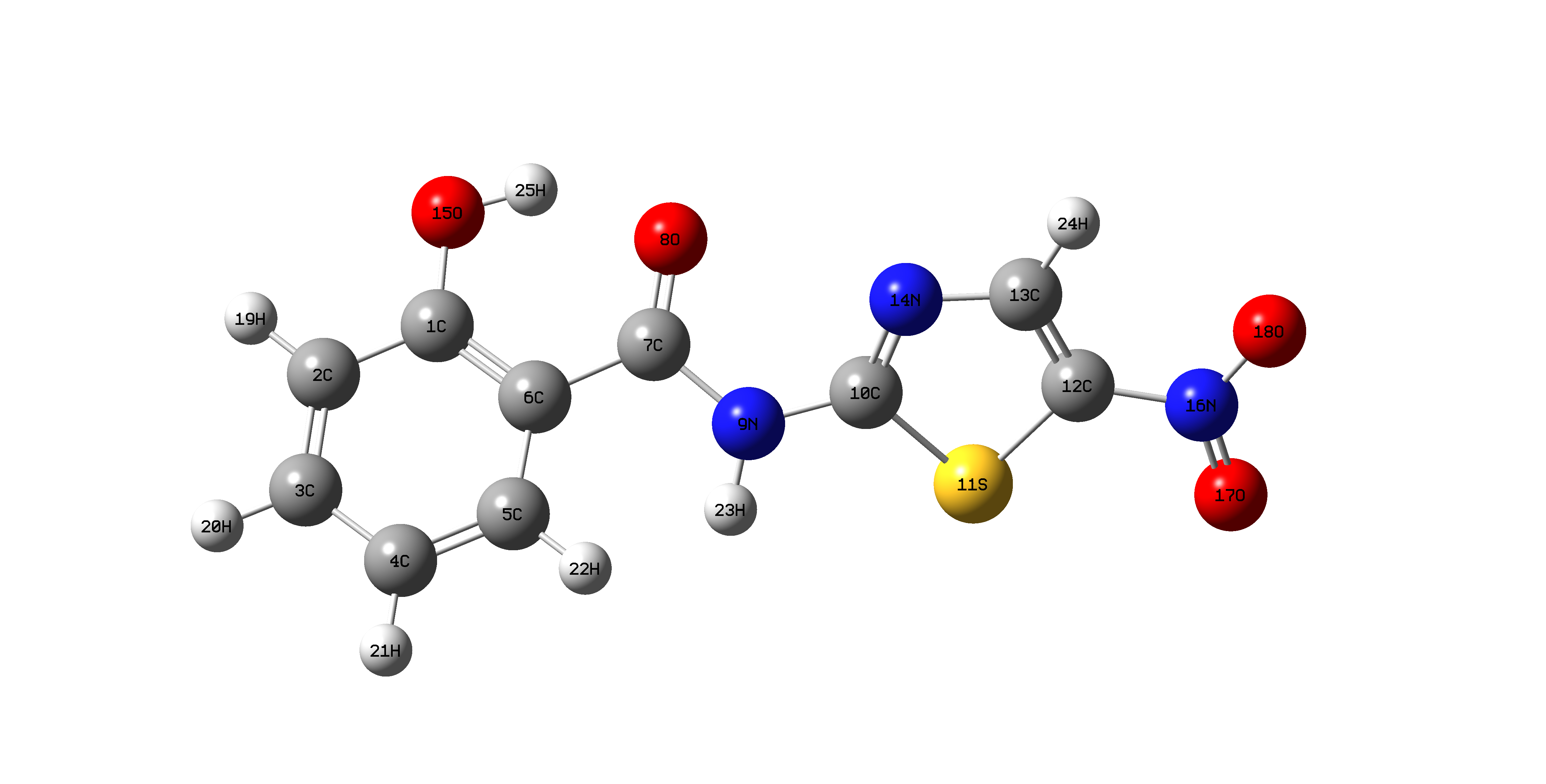

Supplement: Supplementary file 1 [file ijms-26-11578-s001.zip › TIZ/OPTIMIZED 2.png]

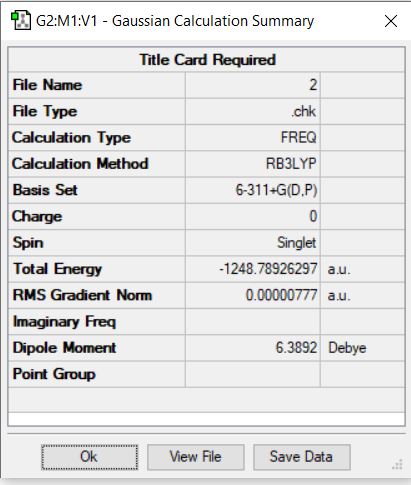

Supplement: Supplementary file 1 [file ijms-26-11578-s001.zip › TIZ/SUMMARY.JPG]
